# Tumor Microenvironment in Prostate Cancer: Toward Identification of Novel Molecular Biomarkers for Diagnosis, Prognosis, and Therapy Development

**DOI:** 10.3389/fgene.2021.652747

**Published:** 2021-03-26

**Authors:** Hisham F. Bahmad, Mohammad Jalloul, Joseph Azar, Maya M. Moubarak, Tamara Abdul Samad, Deborah Mukherji, Mohamed Al-Sayegh, Wassim Abou-Kheir

**Affiliations:** ^1^Department of Anatomy, Cell Biology and Physiological Sciences, Faculty of Medicine, American University of Beirut, Beirut, Lebanon; ^2^Arkadi M. Rywlin M.D. Department of Pathology and Laboratory Medicine, Mount Sinai Medical Center, Miami Beach, FL, United States; ^3^Department of Internal Medicine, Division of Hematology-Oncology, American University of Beirut Medical Center, Beirut, Lebanon; ^4^Biology Division, New York University Abu Dhabi, Abu Dhabi, United Arab Emirates

**Keywords:** prostate cancer, tumor microenvironment, reactive stroma, inflammation, biomarker

## Abstract

Prostate cancer (PCa) is by far the most commonly diagnosed cancer in men worldwide. Despite sensitivity to androgen deprivation, patients with advanced disease eventually develop resistance to therapy and may die of metastatic castration-resistant prostate cancer (mCRPC). A key challenge in the management of PCa is the clinical heterogeneity that is hard to predict using existing biomarkers. Defining molecular biomarkers for PCa that can reliably aid in diagnosis and distinguishing patients who require aggressive therapy from those who should avoid overtreatment is a significant unmet need. Mechanisms underlying the development of PCa are not confined to cancer epithelial cells, but also involve the tumor microenvironment. The crosstalk between epithelial cells and stroma in PCa has been shown to play an integral role in disease progression and metastasis. A number of key markers of reactive stroma has been identified including stem/progenitor cell markers, stromal-derived mediators of inflammation, regulators of angiogenesis, connective tissue growth factors, wingless homologs (Wnts), and integrins. Here, we provide a synopsis of the stromal-epithelial crosstalk in PCa focusing on the relevant molecular biomarkers pertaining to the tumor microenvironment and their role in diagnosis, prognosis, and therapy development.

## Introduction

Prostate cancer (PCa) is the most frequent malignancy affecting men and the second leading cause of cancer-related deaths among males globally (Siegel et al., 2021). While tumors with low pathological grade – that are confined to the prostate at the time of diagnosis – are usually cured, others with advanced “International Society of Urological Pathology (ISUP)” grade groups and metastasis convey a poorer prognosis (Martin et al., 2011). Indolent prostatic neoplastic lesions may be asymptomatic or present with lower urinary tract symptoms (LUTS). In contrast, advanced tumors may cause more severe symptoms related to multi-organ spread such as vertebral fractures or spinal cord compression (Merriel et al., 2018). Androgens play a key role in the pathogenesis of PCa; therefore, treatment modalities altering androgen receptor signaling pathways are essential (Schrecengost and Knudsen, 2013). Gonadal androgen deprivation therapy is the standard of care for disseminated disease. However, despite initial effectiveness of ADT, resistance to therapy will inevitably occur resulting in castration-resistant prostate cancer (CRPC; Polotti et al., 2017).

Epithelial cells are not the only players contributing to PCa tumorigenesis; tumor microenvironment consisting of immune and non-immune cells also play a pivotal role in pathogenesis and disease progression (Shiao et al., 2016). Indeed, prostatic oncogenesis involves cross-talk between epithelial cells and the surrounding stroma *via* a sequence of intrinsic cellular changes and microenvironment alterations. Multiple biomarkers of stromal activity have been implicated in PCa pathogenesis (Filella et al., 2018). The tumor microenvironment is a wide interlinked niche encompassing mesenchymal/stromal stem cells, endothelial cells, fibroblasts, myofibroblasts, immune cells, and neural crest cells, among others, all of which secrete factors such as chemokines, cytokines, extracellular matrices, and matrix-degrading enzymes (Hagglof and Bergh, 2012). Due to the interaction between prostatic epithelial cells and the tumor microenvironment, the surrounding stromal agents undergo complex changes that pilot disease severity, metastatic ability, and resistance to conventional therapies (Hagglof and Bergh, 2012). In the light of the essential role the tumor microenvironment plays in PCa, identification of novel biomarkers for the stromal activity is crucial in the management process. In this review, we elaborate on the epithelial-stromal interplay emphasizing on pertinent biomarkers of the stromal activity and their role in disease diagnosis, prognosis, and therapy development (Table 1).

**Table 1:** Table summarizing relevant molecular biomarkers pertaining to the tumor microenvironment and their role in diagnosis, prognosis, and therapy development in prostate cancer (PCa).

Biomarker	Tumor relevance	Compartment	Source
Collagen type VII	Increased expression in advanced PCa	ECM	Nagle et al., 1995; Bussemakers et al., 2000
*MMP-1, −2, −7, −9, MT1-MMP*	Increased expression indicates cancer progression and metastatic potential	ECM	Gong et al., 2014
*BTF3*	Overexpression enhances metastatic and self-renewal abilities	CSCs	Hu et al., 2019
*rBC2LCN*	Expression confers sluggish proliferation, enhanced cell motility, resistance to therapy, and anchorage-independent growth	CSCs	Mawaribuchi et al., 2019
*Skp2*	Increased invasiveness by cellular epithelial-to-mesenchymal transition	CSCs	Simeckova et al., 2019
*BMI1, Sox2*	Castration resistance and disease recurrence	CSCs	Yoo et al., 2019
*ALDH1A1, ALDH1A3*	Castration resistance	CSCs	Federer-Gsponer et al., 2020
MUC1-C protein	Impedes the androgen receptor and *p53* signaling pathway and increases expression of the Yamanaka factors	CSCs	Papadopoulos et al., 2001
MUC1-C protein	Stimulates angiogenesis	CSCs	Yasumizu et al., 2020
Tumor associated macrophages	Increased expression correlates with shorter median cancer specific survival time and poor clinical outcomes	Inflammatory cells	Lissbrant et al., 2000
Mast cells	Production of *FGF-2* driving tumoral progression into CRPC	Inflammatory cells	Johansson et al., 2010
*TNF*	Shuts down angiogenesis inducing tumor regression and activates transcription factors promoting tumor growth Elevated serum levels established in hormone refractory states	Cytokines	Mizokami et al., 2000; Hu et al., 2004
*NF-κB*	Alters the expression of *c-myc, cyclin-D1*, and *IL-6* and enhances production of *VEGF* and *IL-8*	Transcription factors	Suh and Rabson, 2004; Lee et al., 2007
IL-6	Increased serum levels relate to metastatic or hormone refractory tumors indicating a poorer prognosis	Interleukins	Adler et al., 1999; Drachenberg et al., 1999
IL-8	Increased concentrations enhance metastasis and progression into docetaxel-refractory CRPC	Interleukins	Inoue et al., 2000
Stromal derived factor-1 (*CXCL12*)	Interaction with CXCR4 (CXCL12 receptor) ensues pro-angiogenesis and metastasis	Chemokines	Sun et al., 2003; Jung et al., 2006
miR-21, miR-222, miR-125B	Mediators of progression and maintenance of CRPC	Nucleic acids	Scott et al., 2007; Mercatelli et al., 2008; Ribas et al., 2009
miR-1301p	Upregulates expression of stemness pathways and decreases the expression of Wnt pathway inhibitors	Nucleic acids	Song et al., 2018
*CUL4B*	Control stemness properties by utilizing miR200b/c to target *BMI1*	ECM	Jiao et al., 2019
LncRNA H19, LncRNA HOTAIR	Maintenance of PCa stemness	Nucleic acids	Huang et al., 2017
*PTX3*	Increased prostate tissue expression and elevated serum levels correlate with PCa development	Immune mediators	Stallone et al., 2014
*VEGF*	Unregulated expression in response to *HIF-1* inducing endothelial proliferation, vascular permeability, carcinogenesis, and site-specific metastasis to bone tissue. Induces expression Bcl-2 and A-1 anti-apoptotic proteins to promote tumor growth	Growth factors	Cvetkovic et al., 2001; Chen et al., 2004; Sterling et al., 2011; Roberts et al., 2013
*FGF1, FGF2, FGF6, FGF8*	High expression of FGF2 and FGF2 receptors aids in stimulating fibroblasts and promoting angiogenesis leading to advanced androgen-independent PCa with low survival rates. ECM degradation and angiogenesis by plasmin-plasminogen activator and *MMP* upregulation	Growth factors	Nakamoto et al., 1992; Dell’Era et al., 1999; Polnaszek et al., 2003; Kwabi-Addo et al., 2004; Akl et al., 2016
*TGF-β*	*TGF-β* induces angiogenesis by stimulating the expression of *VEGF* and *CTGF* in epithelial cells and fibroblasts. Loss of *TGF-β-I* receptor is associated with high grade tumors, higher clinical stage, and reduced 4-year survival rate	Growth factors	Pertovaara et al., 1994; Kim et al., 1996; Shimo et al., 2001
*COX-2*	Mediates hypoxia induced angiogenesis through the production of prostaglandins. Elevated COX-2 levels correlate with PCa having high gleason scores	Immune mediators	Fujita et al., 2002; van Moorselaar and Voest, 2002; Wang et al., 2005
*PDGF*	*PDGF* binds *PDGFRα* which is highly expressed in primary PCa activating the Akt pathway promoting tumor progression and bone metastasis. Stimulates angiogenesis by upregulating *VEGF-A*	Growth factors	Chott et al., 1999; Laschke et al., 2006; Russell et al., 2009; Chen et al., 2013
*Ang-1*	*Tie2* receptor ligand, a pro-angiogenic factor, enhances tumor growth and induced sprouting angiogenesis	Angiogenesis stimulating factor	Satoh et al., 2008
*EGF*	Downregulates miR-1 and activates *TWIST1* causing accelerated PCa bone metastasis	Angiogenesis stimulating factors	Ching et al., 1993; Chang et al., 2015
*TGF-α*	Downregulates miR-1 and activates *TWIST1* causing accelerated PCa bone metastasis	Angiogenesis stimulating factors	Ching et al., 1993; Chang et al., 2015
Scatter factor/hepatocyte growth factor	Stimulates angiogenesis	Angiogenesis stimulating factors	Zhu and Humphrey, 2000
Tissue factor	Stimulates angiogenesis	Angiogenesis stimulating factors	Abdulkadir et al., 2000
*ACK1/TNK2*	Regulates progression into CRPC	CSCs	Mahajan et al., 2018
Calcitonin	Enhanced adherence to collagen and expression of *CD133* and *CD44* potentiating tumorigenesis and metastatic abilities	ECM	Aldahish et al., 2019
*THBS4*	Stimulates PI3K/Akt pathway to advance tumor progression	Glycoproteins	Hou et al., 2020
Neurotrophic growth factors	Initiate tumor innervation	Brain-derived progenitor cells	March et al., 2020
Skeletal/smooth muscle cells	Increase IL-4 and IL-13 secretion, overexpress annexin A5, and aid in cancer cell fusion	Muscle cells	Uygur et al., 2019

## Stromal Compartment in PCa

**Figure 1:**
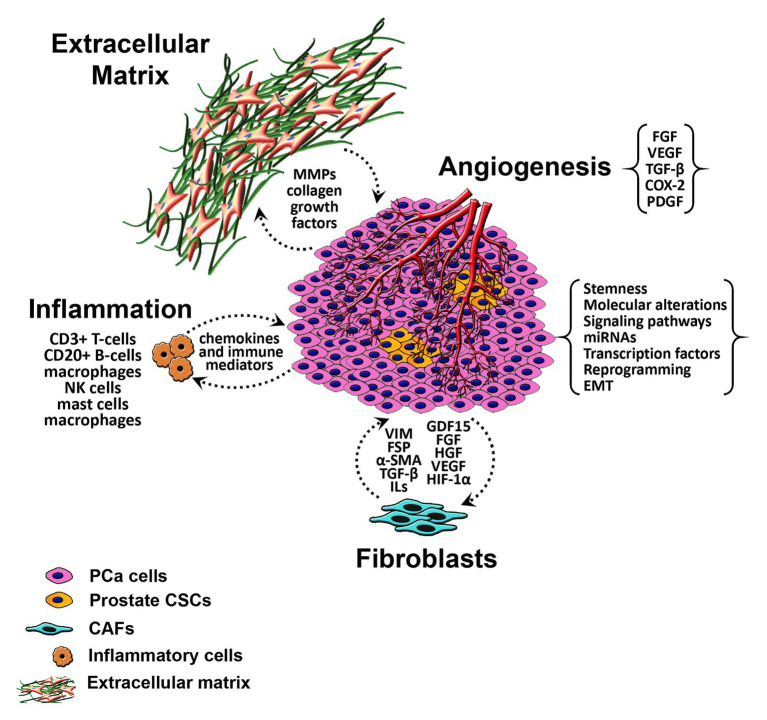
Schematic diagram of the tumor microenvironment in PCa. α-SMA, alpha smooth muscle actin; CAFs, cancer associated fibroblasts; CSCs, cancer stem cells; COX-2, cyclooxygenase-2; ECM, extracellular matrix; EMT, epithelial-to-mesenchymal transition; FGF, fibroblast growth factor; FSP, fibroblast specific protein; GDF15, growth differentiation factor 15; HGF, hepatocyte growth factor; ILs, interleukins; miRNAs, micro-RNAs; MMP, matrix metalloproteinases; NK cells, natural killer cells; PCa, prostate cancer; PDGF, platelet-derived endothelial cell growth factor; TGF-β, transforming growth factor beta; VEGF, vascular endothelial growth factor.

The prostatic stromal microenvironment includes multiple components that are anatomically and physiologically pertinent for a normal function of the gland. Alteration in some of these stromal factors plays a role in the development and progression of PCa (Figure 1). The process of epithelial neoplastic transformation in the prostate is not independent from its surrounding; molecular alteration within the cells besides factors of the microenvironment plays a significant role. Indeed, prostatic tumoral growth and metastasis relies on the interplay between neoplastic cells and the constituents of the stroma (Torrealba et al., 2017). Fibroblasts are key players in the prostatic stroma. They maintain the integrity of epithelial cells by constantly remodeling and interacting with different elements within the organ (Gabbiani, 2003). Fibroblasts participate in extracellular matrix (ECM) formation through secreting collagen type-I and -III and permit tissue repair *via* controlled granulation tissue formation and transition into myofibroblasts (MFB). In prostatic neoplastic transformation, stromal smooth muscle cells are replaced by specialized fibroblasts termed cancer-associated fibroblasts (CAF). Cancer stroma also prompts an increase in the expression of fibroblast-specific markers such as vimentin, fibroblast specific protein (FSP), and alpha smooth muscle actin (α-SMA), and a decrease in the expression of desmin (Hagglof and Bergh, 2012). CAFs present a major source of different factors inducing angiogenesis and altering the ECM components such as transforming growth factor beta (TGF-β), interleukin-6 (IL-6), growth differentiation factor 15 (GDF15), fibroblast growth factor (FGF), hepatocyte growth factor (HGF), hypoxia inducible factor 1 alpha (HIF-1α), and vascular endothelial growth factor (VEGF; Ishii et al., 2011; Levesque and Nelson, 2018). The proposed activity of CAF in response to its interaction with tumor cells leads to the formation of an uncontrolled “reactive stroma” stimulating cancer cell proliferation, aggressiveness, and affecting response to treatment (Sahai et al., 2020).

Prostatic tumorigenesis is undoubtedly dependent, to a certain extent, on angiogenesis upregulation. Formation of blood vessels is a crucial phenomenon to support cancerous cell viability and growth (Bates et al., 2008). In normal prostatic tissue, there exists a balanced interaction between endothelial cells, pericytes, and smooth muscle cells. However, tumor vasculature is characterized by aberrant formation of immature leaky blood vessels that lack covering pericytes (Bergers and Benjamin, 2003). Interaction between tumor cells and surrounding stromal endothelial cells promotes an “angiogenic switch” by increasing pro-angiogenic factors such as VEGF (Bergers and Benjamin, 2003). Zhao et al. (2018) demonstrated that endothelial cells are a main component of the tumor microenvironment for their role in stimulating metastatic activity *via* suppressing both androgen receptor (AR) expression and transcriptional activity, hence proposing that their inhibition could hinder PCa progression.

Immune cells are normal residents of a healthy prostatic tissue and play a protective role against infiltrating pathogens (Hussein et al., 2009). However, histological studies have shown that high-grade PCa is associated with increased stromal immune cell infiltrates with difference in cellular types according to tumor stage (Gurel et al., 2014). Ongoing stressors such as direct infection, urinary reflux, high fat diet, and estrogens influence the initiation of a chronic inflammatory state within the prostate (De Marzo et al., 2007). Amidst persistent inflammation, the stromal compartment witnesses an influx of multiple immune cells such as CD3+ T-cells, CD20+ B-cells, macrophages, natural killer (NK) cells, mast cells, and macrophages (Shiao et al., 2016). Inflammatory cells produce high amounts of cytokines and chemokines, mainly tumor necrosis factor (TNF), Nuclear Factor Kappa B (NF*κ*B), IL-6, IL-8, and VEGF, to name a few. These proteins and others play a role in controlling angiogenesis, cellular proliferation, and inflammation. They direct the transition toward malignant phenotype in PCa (Worthington et al., 2012). The participation of inflammation in prostatic malignancy allowed the utilization of novel anti-inflammatory drugs in preventing and possibly treating PCa (Stark et al., 2015).

The tight interlocking ECM surrounding prostate epithelial cells contains collagenous fibers and multiple non-collagenous proteins such as fibronectins, bone sialoproteins, osteocalcins, cadherins, osteonectins, and vitronectins (Kiefer and Farach-Carson, 2001). This ECM acts as a scaffold to maintain normal functioning of different cells within the organ. As neoplastic process hits in and metastatic progression ensues, the expression of different extracellular residents is upregulated, downregulated, or lost. Advanced PCa is associated with a decrease in the expression of collagen type VII and an increase in the production of bone sialoproteins (Nagle et al., 1995; Bussemakers et al., 2000). It is evident that metastatic progression is dependent on a breach to the barrier formed by the ECM. Multiple proteases and degrading proteins must be produced by cancerous cells for invasion and metastasis to occur (Stamenkovic, 2000). Matrix metalloproteinases (MMP) are zinc-dependent peptidases with wide affinity to ECM proteins such as collagens, fibronectins, and laminin (Cooper et al., 2003). As PCa develops, MMPs, such as MMP-1, -2, -7, -9 and MT1-MMP, are increasingly manifested within the stroma and in the circulation, indicating the potential role these molecules can play as prognostic tools (Gong et al., 2014). Yet, MMP inhibiting drugs, batimastat, and marimastat, failed to show efficacy in phase III clinical trials despite being theoretically promising in targeting PCa (Coussens et al., 2002).

## PCa Microenvironment and Cancer Stem/Progenitor Cell Markers

Although the existence of prostate stem cells was first reported in the 1980s (English et al., 1987), the notion that PCa may be derived from CSCs started to become widely accepted. This has driven a rising wave of scientific interest aiming at characterizing these stem cells (Abou-Kheir et al., 2010; Leao et al., 2017). Their unique ability of self-renewal, pluripotency, plasticity, and ability to restore full tumor heterogeneity propelled a new era of therapeutics and diagnostics in which the need to fully understand how to detect prostate CSCs and identify their potential markers has become crucial (Moltzahn and Thalmann, 2013; Bahmad et al., 2018, 2020a). The prostate CSC’s resistance to therapy often extends to include radiotherapy and this could be attributed to complex mechanisms such as a hypoxic microenvironment, improved DNA repair, epithelial-to-mesenchymal transition (EMT; Tsai et al., 2018; Cheaito et al., 2019), amplified intracellular reactive oxygen species scavenging, autophagy, and initiation of anti-apoptotic signaling (Tsao et al., 2019). It is important to note that prostate CSCs constitute a marginal percentage of the total tumor mass, with most of them being in proximal regions of the prostatic ducts. These stem cells reside in niches with complex microenvironments that closely intermingle with host cells. Although it has been widely accepted that prostate CSCs are mostly located in the basal cell compartment, controversy and ongoing research are still rising when it comes to the existence of such cells in the luminal compartment as well (Adamowicz et al., 2017; Xin, 2019). The dramatic drop in the survival rates of patients with recurrent or metastatic tumors was one of the main drivers of the hunt for CSCs in the prostate.

Harris et al. showed that a multitude of CSC markers is heavily involved in PCa development and progression, besides resistance to therapy and metastatic colonization and growth (Harris and Kerr, 2017). Extracellular CSC markers that have been implicated in PCa but are not always specific to this cancer type *per se* include *CD117/c-kit, CD133, CD44, α2β1 integrin, α6 integrin, CXCR4, E-cadherin, EpCAM, Cytokeratin 5, PSA, ABCG2, Trop2, AR variant 7*, and *CD166/ALCAM* (Stefano et al., 2018). Intracellular markers include *ALDH1, TG2*, and *EZH2*. One must not forget the classical stemness markers such as *Sox2, Oct3/4, Nanog, c-myc*, and *Klf4* (Harris and Kerr, 2017). Having mentioned all these markers, it would be sound to highlight the most recent advances in the characterization of prostate CSCs. Hu et al. recently identified basic transcription factor 3 (*BTF3*) expression to be highly associated with stemness traits. For instance, the metastatic potential and self-renewal abilities were reduced by *BTF3* loss and enhanced by *BTF3* overexpression. The postulated mechanism revolves around the theory that *BTF3* could stabilize *BMI1*, which is a key regulator of prostate CSC self-renewal. The investigators further demonstrated that *BTF3* may highly predict a poor prognosis and can be, therefore, used to risk-stratify patients (Hu et al., 2019). Mawaribuchi et al. revealed that the recombinant lectin *rBC2LCN* may be potentially used as a CSC marker in PCa. In fact, the subpopulation of PC-3 *rBC2LCN*-positive cells exhibited a sluggish proliferation, enhanced cell motility, resistance to therapy, and anchorage-independent growth, all of which are features of CSCs (Mawaribuchi et al., 2019).

Simeckova et al. assessed the expression of *Skp2* which is a key component of SCF E3 ubiquitin ligase and is frequently overexpressed in PCa and other neoplasms. The study revealed that Skp2 was highly expressed in PCa cells with a stem cell-like and mesenchymal profile when compared to epithelial cells. It is worth noting here that a transition from epithelial to non-epithelial/mesenchymal phenotype was previously shown to be linked to the invasiveness seen in cancer stemness. The stemness phenotype was reduced in cells with a silenced *Skp2*. Moreover, *Skp2* downregulation diminished the subpopulation of *CD44*+/*CD24*− PCSC, which further supports the notable involvement of Skp2 in PCa stemness (Simeckova et al., 2019). Using lineage retracing, Yoo et al. identified a subpopulation of *Bmi1+ Sox2+* CRPC cells as a potential source of *in vivo* disease recurrence. These findings could encourage the targeting of specific culprit subpopulations for therapeutic purposes (Yoo et al., 2019). Using paired hormone-naïve and castration-resistant samples, Federer-Gsponer et al. (2020) similarly highlighted another stemness-associated marker pattern that could be associated with castration resistance. A very interesting finding was a unique pattern of mutual exclusivity between *ALDH1A1* and *ALDH1A3* expression. To note, this pattern was found in public datasets at the transcriptomic level. Therefore, further studies would be of utmost importance to decipher this pattern and to reach a better understanding of the differential contribution of these markers.

A different category of markers that are of interest are stromal markers. For instance, Mahal et al. revealed the importance of such markers by conducting a genome-wide analysis of radical prostatectomy samples. After correlating expression scores with classical stromal genes and with other key stromal markers (such as *CAV1, TAGLN*, and *VIM*), *CD3* and *CD4* markers and basal activity, the researchers concluded that the top 10% of stromal expression was associated with high genomic risk scores (Decipher ≥ 0.6), high Cancer of the Prostate Risk Assessment-Postsurgical (CAPRA-S) scores, Gleason 9 to 10 disease, and an increased risk for metastasis (hazard ratio, 2.35; 95% CI, 1.37–4.02; *p* = 0.001). Moreover, a higher stromal infiltration score was associated with a reduced expression of DNA repair genes and increased radiation sensitivity genomic scores (Mahal et al., 2020). From another perspective, Yasumizu et al. focused on the MUC1-C protein which is highly expressed in castration resistant neoplasms and neuroendocrine PCa. In androgen-dependent PCa cells, the upregulation of *MUC1-C* hampers both the androgen receptor and p53 signaling pathways. On the other hand, it also leads to the expression of *OCT4, KLF4, MYC*, and *SOX2* (known as the Yamanaka factors), thus leading to an enhanced stemness. Therefore, therapeutically speaking, MUC1-C could be used as a therapeutic target against PCa stemness (Yasumizu et al., 2020).

Although the current advances are partially focused on liquid biopsies as a minimally invasive procedure to characterize prostate CSCs, perhaps a breakthrough in the field of marker identification would be to shift from PCa cell lines to patient-derived tumors which can provide a better understanding of metastatic and treatment resistance processes. Relying on patient-derived organoids that can replicate the molecular, biochemical, and structural diversity of the original tumor may further support the current research efforts and overcome the limitations of maintaining an *in vitro* luminal phenotype (Li and Shen, 2019).

## Stromal-Derived Mediators of Inflammation in PCa

Inflammatory cascades have been immensely discussed in the tumorigenesis of PCa (Dennis et al., 2002; Bilani et al., 2017). Inflammation in the prostate can be caused by infectious pathogens such as Neisseria Gonorrhea and Chlamydia Trachomatis or noninfectious triggers characterized by urinary reflux, diet, or autoimmune processes (Kirby et al., 1982; Dennis et al., 2002). A meta-analysis by Dennis et al. (2002) established an increased risk (Odds Ratio = 1.6–1.8) of PCa in men who have previously suffered from infectious prostatitis. Chronic inflammation within normal prostate tissue was evidently linked to biopsy proven high-grade prostatic malignancies (Gurel et al., 2014). In this context, chronic inflammation turns the prostatic microenvironment into a milieu rich in pro-inflammatory cytokines, chemokines, growth factors, and immune cells concurrently interacting with each other and with epithelial cells to induce uncontrolled proliferation and angiogenesis within the organ (Steiner et al., 2002). Cellular inhabitants within the gland are altered by factors driving the influx of innate and adaptive immune mediators (Condeelis and Pollard, 2006; Halin et al., 2011).

The tumor microenvironment witnesses migration of monocytes, mast cells, dendritic cells, NK cells, polymorphonuclear cells, and lymphocytes (Lissbrant et al., 2000; Karja et al., 2005; Johansson et al., 2010). Lissbrant et al. (2000) demonstrated that an increase in the volume density and cell profile area of tumor associated macrophages (TAM), derived from infiltrating monocytes, reflect a shorter median cancer specific survival time and poorer clinical outcomes. Mast cells located within the stroma contributed to the expansion of the tumor by providing FGF-2. Moreover, mast cell recruitment into the peritumoral stroma was specifically observed with the progression of PCa into CRPC, indicating that mastocytes are independent markers of poor prognosis (Johansson et al., 2010). Different inflammatory cells within the tumor microenvironment communicate with epithelial cells in many ways, one of which is by secreting oxygen and nitrogen species. For example, hydrogen peroxide (H_2_O_2_) and nitric oxide (NO) interact with genetic material of the epithelium causing permanent genomic alterations driving their chaotic proliferation (Moldogazieva et al., 2019). This interplay has been invoked to ignite the inflammation-carcinoma cascade in PCa (Dakubo et al., 2006; Kumar et al., 2008).

In the setting of chronic inflammation within the prostate, atrophy of the epithelium, glandular structures, and surrounding stroma is established in a focal or diffuse fashion. Focal atrophy within the gland preferentially occurs in the outer peripheral zones (McNeal, 1988). According to an immunohistological study done by De Marzo et al. (1999), these foci exhibited resident cells with a counterintuitive increased staining for glutathione S-transferase pi gene (GSTP1) and Bcl-2 and decreased staining for cyclin-dependent kinase inhibitor p27^Kip1^, indicating a highly proliferative propensity. This interesting inflammatory-driven phenomenon was termed “proliferative inflammatory atrophy (PIA)” and is formulated to be a well-built bridge connecting prostatic inflammation and carcinogenesis.

Inflammatory conditions trigger immune cell and stromal release of multiple mediators within the tumoral microenvironment. TNF, a multifunctional cytokine, plays a paradoxical role in the pathogenesis of tumors, including PCa (Ferrajoli et al., 2002). Based on stimuli, this cytokine can shut-down angiogenesis inducing tumor regression (Davis et al., 2011) or promote cellular proliferation and tumor growth through activation of transcription factors such as NF*κ*B (Hu et al., 2004). Elevated serum levels of TNF were established in PCa hormone refractory states suggesting a promising characteristic as a biomarker for CRPC (Mizokami et al. , 2000). PCa microenvironment expresses high concentrations of NFκB (Suh and Rabson, 2004); activation of this transcription factor alters the expression of cell cycle orchestrators such as c-myc, cyclin-D1, and IL-6 and enhances production of angiogenic factors such as VEGF and IL-8 (Lee et al., 2007). Another cytokine, IL-6, was described as a key effector in PCa pathogenesis. It is produced by the tumor microenvironment cells and acts on prostatic epithelial cells in a paracrine way to modulate tumor progression (Yu et al., 2019). As evident by several studies, increased serum levels of IL-6 correlate with metastatic or hormone refractory PCa (Adler et al., 1999; Drachenberg et al., 1999). The emerging importance of IL-6 in PCa outlines its significance as an indicator of poor prognosis. Therapeutic rationales targeting IL-6 such as Siltuximab were investigated in patients with metastatic CRPC refractory to standard treatment but failed to improve disease outcomes (Fizazi et al., 2012). Additionally, increased IL-8 concentrations within PCa microenvironment are consistent with higher cancerous cell adherence to the endothelium, thus enhancing tumor angiogenesis and metastatic spread (Inoue et al., 2000). The aforementioned interleukin desensitizes PCa cells to androgens and facilitates progression into docetaxel-refractory metastatic CRPC (Inoue et al., 2000). Hence, agents decreasing IL-8 levels such as naphthalimide aid in treating advanced forms of this malignancy (Mijatovic et al., 2008).

The main chemokine receptors investigated in the pathogenesis of PCa are CXCR4, CXCR6, and CXCR7 (Thobe et al., 2011). Of particular relevance to PCa growth and dissemination is the biological axis formed by the CXCL12 chemokine, also known as the stromal-cell derived factor-1 (SDF-1), and its receptor CXCR4 (Petit et al., 2007). CXCL12 and CXCR4 are highly expressed in malignant prostatic tissue evidently aiding in trafficking hematopoietic stem cells and endothelial precursors which have direct or indirect pro-angiogenic properties into the tumor microenvironment (Sun et al., 2003). Moreover, CXCL12 enhances adhesion to bone marrow endothelial cells and allows trans-endothelial migration of prostatic malignant cells expressing CXCR4 toward areas of high CXCL12 concentration, such as bone tissue (Jung et al., 2006). This interaction delineates a crucial step in PCa site-specific metastasis.

Periprostatic adipose tissues (PPAT) are metabolically active sites that contribute to prostate tumorigenesis through paracrine secretion of growth hormones, cytokines, and adipokines into the tumor microenvironment (Mistry et al., 2007; Finley et al., 2009). Finley et al. (2009) proved that PPAT induces PCa by increasing secretion of IL-6. Also, Ribeiro et al. (2012) revealed that PPAT produces MMPs that degrade the prostatic ECM facilitating tumoral dissemination and progression into metastatic PCa. Therefore, targeting signaling pathways and secreted factors within the PPAT could be useful in limiting PCa advancement (Sacca et al., 2019).

Micro-RNAs (miRNAs) are non-coding RNAs that alter gene expression at the post-transcriptional level affecting cell differentiation and proliferation. Inflammation induces damage to DNA material within the prostate form new miRNA recognition sites, and thus increases miRNA activity and allows role-playing as oncogenes or tumor-suppressors. Alterations in miRNA expression or activity are associated with tumor development and progression in various organs, including the prostate (Calin et al., 2004). For instance, Zhu et al. (2020) demonstrated that miRNA-671-5p promote PCa development and metastasis by suppressing NFIA/CRYAB axis. Also, in PCa, elevated levels of miRNA-21, miRNA-222, and miRNA-125B are established and serve as a mediator in progression and maintenance of CRPC (Scott et al., 2007; Mercatelli et al., 2008; Ribas et al., 2009). miRNA’s inherent stability, easy methods for quantification (qRT-PCR), and relatively easy detection within bodily fluids (such as serum, saliva, semen, and urine) make them promising tools to improve the accuracy of current available diagnostic modalities in PCa such as prostate-specific antigen (PSA), digital rectal exam (DRE), and sonography tests (Bidarra et al., 2019).

Huang et al. (2017) highlighted in a review the role of long non-coding RNA such as LncRNA H19 and LncRNA HOTAIR in maintaining PCa stemness. Song et al. investigated the role of miR-1301-3p in PCa progression. It was shown that miR-1301-3p induced the growth of CSCs and upregulated the expression of stemness pathways such as *MMP2, OCT4, SOX2, NANOG, c-MYC, CD44*, and *KLF4*. In fact, miR-1301-3p decreases the expression of Wnt pathway inhibitors, SFRP1 and GSK-3β by directly binding to their 3′ untranslated regions. It is worth noting here that TOP/FOP luciferase assays also confirmed that it is an activator of the Wnt pathway. Furthermore, after examining the expression of miR-1301-3p in tissues, it was shown that there was a negative correlation between miR-1301-3p levels and GSK-3β and SFRP1 expression (Song et al., 2018). Another example of microRNAs involvement would be Cullin 4B. It is a member of the ubiquitin E3 ligase that has a significant expression in many malignancies. Jiao et al. (2019) demonstrated that CUL4B has a role in controlling stemness properties of PCa cells by targeting BMI1 *via* miR200b/c.

Pentraxin 3 (PTX3), a member of the pentraxin family of proteins is an integral arm of the innate immunity responsible for continuous cellular proliferation, angiogenesis, and insensitivity to apoptosis (Garlanda et al., 2005; Baruah et al., 2006). Stallone et al. (2014) significantly linked an increased prostate tissue expression and elevated serum levels of PTX3 to development of PCa. This finding sets out PTX3 as a potential biomarker linking prostatic inflammation and carcinogenesis.

## Regulators of Angiogenesis in PCa

Angiogenesis is a dynamic physiological process for the formation of new blood vessels either by sprouting angiogenesis by which the vascular endothelium sprouts from pre-founded capillaries or by a non-sprouting mode whereby the pre-existing capillaries divide into new daughter vessels (Wang et al., 2019). Angiogenesis is pivotal not only for chronic inflammation, tumor growth, and metastasis but also for prostate embryonic development, tissue repair, fertility, and wound healing (Folkman, 1971; Wang et al., 2019). In PCa, higher microvessel density is accompanied by a poor prognosis, tumor survival, progression, and metastasis (Vartanian and Weidner, 1995; Strohmeyer et al., 2000; Li and Cozzi, 2010). The angiogenic switch is regulated by many factors including the angiogenic inducers such as angiopoietins, CCL2, EGFL6, endothelins, FGF, HIF1, IGF1, MMPs, PDGF, TGF, VEGF, and endogenous inhibitors like angiostatin, endostatin, TSP1, and PAI2 (Wang et al., 2019). Angiogenic inhibitors have been studied in the treatment of PCa but have failed to provide clinical benefits (Melegh and Oltean, 2019). It is postulated that resistance to anti-angiogenic drugs is due to angiogenic pathway redundancy (Pavlakovic et al., 2001), loss of the tumor suppressor protein phosphatase and tensin homolog (PTEN; Carver et al., 2011), and the presence of multiple pro- and anti-angiogenic VEGF splicing isoforms (Bates et al., 2002). Nevertheless, a careful analysis of the potential constraints that hampered the success of anti-angiogenic drug trials could perhaps direct future trials to a more adequate path. For instance, using progression-free survival and PSA response may not be suitable for anti-angiogenic drugs given the exceptionally lengthy survival of PCa patients. Therefore, there will be a need for an intensive search for markers of anti-neoplastic activity in general and anti-angiogenic activity specifically. Perhaps modern imaging techniques could also help in clinically tracking the effect of such drugs. Since men with PCa present at an older age than other cancer patients, a closer attention should also be paid to the safety profiles of such drugs, knowing that this constitutes a major bottleneck when it comes to achieving a trial’s success. Finally, combining anti-angiogenic drugs with other drugs of different classes may potentially open a door for more promising clinical results (Bilusic and Wong, 2014).

### Vascular Endothelial Growth Factor

Vascular endothelial growth factor, an endothelial cell-specific mitogen, induces angiogenesis by inducing endothelial cell proliferation and vascular permeability (Campbell, 1997). Activation of VEGF facilitates the proliferation and migration of endothelial cells to form immature vasculature by inducing the secretion of proteolytic enzymes to degrade both the basement membrane and ECM. Also, VEGF induces the expression of Bcl-2 and A1 anti-apoptotic proteins to promote cell survival (Roberts et al., 2013). In normal prostate tissue, VEGF expression is shown to be either null or minimal (Doll et al., 2001). However, in prostate tumors under hypoxic conditions, VEGF exhibits vast upregulation in response to hypoxia-inducible factor 1 (HIF-1; Cvetkovic et al., 2001), and it is expressed beyond the basal layer, including neoplastic secretory cells (Kollermann and Helpap, 2001). On the other hand, upregulated VEGF expression correlates with PCa progression, tumor stage, and clinical outcome (Borre et al., 1998; Amankwah et al., 2012; Tomic et al., 2012). In a study conducted by Duque et al. (1999), PCa patients with metastasized disease had higher plasma VEGF levels than those with localized disease or healthy controls. Also, VEGF levels were predictive of survival by which patients with urine VEGF level more than 28 pg/ml had shorter survival (10 months) compared to those with VEGF level below 28 pg/ml (Bok et al., 2001). VEGF signal transduction is mediated through VEGF receptors, mainly VEGFR1 and VEGFR2, which are differentially expressed in PCa and benign prostatic hypertrophy (BPH) tissue. Through paracrine actions, VEGF regulates endothelial cell functions and neovascular development, while the expression of both receptors on tumor cells mediates autocrine regulation of tumor cell proliferation (Ferrer et al., 1999). In contrast to other metastases, human prostate metastasis favors an osteoblastic phenotype and bone is the prominent metastatic site for PCa (Cossigny and Quan, 2012; Curtin et al., 2012). The bone microenvironment provides specific conditions that favor PCa bone metastases such as Matrix metallopeptidase 9 (MMP9) which is produced by osteoclasts and induces vascular development in PCa bone metastases (Bruni-Cardoso et al., 2010). VEGF aids in tumor cell recognition of bone and encourages their nesting through regulating metastatic PCa cells migration to fibronectin and bone sialoprotein within the ECM *via* VEGFR-2 (Chen et al., 2004; Sterling et al., 2011). Additionally, VEGF and its cognate receptor VEGFR-2 activate alphaVbeta3 (ɑ_v_β_3_) and alphaVbeta5 (ɑ_v_β_5_) integrins to migrate toward SPARC in bone, thereby promoting tumor growth, neo-angiogenesis, and development of the metastatic tumor (De et al., 2003).

### Fibroblast Growth Factor

Several fibroblast growth factors, including FGF1 (acidic FGF), FGF2 (basic FGF), FGF6, and FGF8, are all expressed in PCa and act as paracrine and/or autocrine growth factors for the PCa cells (Kwabi-Addo et al., 2004). FGF2, mainly synthesized by stromal fibroblasts, acts as a paracrine growth factor for the epithelial PCa cells (Dow and deVere White, 2000; Polnaszek et al., 2003). Low-grade tumors and androgen-dependent PCa cells synthesize and express low amounts of FGF2 mRNA and FGF2 receptors compared to androgen-independent cells (Nakamoto et al., 1992; Kwabi-Addo et al., 2004). FGFs bind to four different transmembrane tyrosine kinase receptors including both FGFR-1 and FGFR-4 that are expressed in PCa cells (Giri et al., 1999; Ropiquet et al., 2000). High expression of FGF2 correlates with a low survival rate for relapsed/refractory cancer patients and contributes to disease progression (Polnaszek et al., 2003; Akl et al., 2016). FGF2 is present in the basal lamina of blood capillaries, at the sites of vessel branching, and in the endothelium of the capillaries of some tumors and stimulates prostate fibroblasts and angiogenesis in an autocrine manner while it exerts paracrine effects on prostate epithelial cell growth (Seghezzi et al., 1998; Ropiquet et al., 2000; Sugamoto et al., 2001). FGF2 promotes ECM degradation and angiogenesis by upregulating plasmin-plasminogen activator (uPA) and MMP (Dell’Era et al., 1999). Also, it binds to ɑ_v_β_3_ integrin to promote endothelial cell adhesion, migration, proliferation, and morphogenesis (Tanghetti et al., 2002). Besides, FGF-induced signaling induces resistance to VEGF receptor signaling for blocking of the VEGF (Casanovas et al., 2005). Taken together, FGF2 is considered as a potent pro-angiogenic agent promoting endothelial cell angiogenesis.

### Transforming Growth Factor β

In benign prostate epithelium, TGF-β acts in paracrine mode to maintain epithelial homeostasis. TGF-β mediates its activity through three receptors, types I, II, and III (van Moorselaar and Voest, 2002). Loss of expression of TGF-β-I and II receptors correlates with poor prognosis in PCa patients. Loss of TGF-β-I receptor associates with high-grade tumors, higher clinical tumor stage, and reduced 4-year survival rate (Kim et al., 1996). Endothelial cells express two distinct type-I receptors: ALK5 and ALK1. TGF-β-I-ALK5 receptor activates the Smad2/3 pathway yielding vessel maturation and angiogenic resolution, while the TGF-β-I-ALK1 receptor antagonizes TGF𝛽/ALK5 responses as it induces Smad1/5 and transcriptional responses related to angiogenesis (Lebrun, 2012). TGF-β induces angiogenesis by stimulating the expression of VEGF and connective-tissue growth factors (CTGF) in epithelial cells and fibroblasts (Pertovaara et al., 1994; Shimo et al., 2001). Together with secretion, and activity of MMP, TGF-β mediates the dissolution of vessels surrounding the tumor as well as the release of endothelial cells from the basement membrane, promoting their migration and invasion (Hagedorn et al., 2001).

### Cyclooxygenase-2

Cyclooxygenase interacts with VEGF to mediate hypoxia-induced angiogenesis (Nicholson and Theodorescu, 2004). Prostatitis that occurs in men above 40 years old, upregulates the expression of cyclooxygenase-2 (COX-2) as well the production of anti-oxidants that causes cellular or genomic damage in the prostate (Hughes et al., 2005; Tkacz et al., 2005). In normal prostate tissue, COX-2 expression is weak, or negative compared to that in PCa tissue (Kirschenbaum et al., 2000; Zha et al., 2004). In a study by Wang et al., prostate tumor cells near areas with chronic inflammation revealed COX-2 expression upregulation. Elevation in COX-2 was associated with PCa tissues having high gleason scores. Also, microvessel density (MVD) correlates positively with COX-2 expression as well as COX-2 expression and angiogenesis in PCa (Wang et al., 2005). COX-2 induces angiogenesis through the production of prostaglandins such as prostaglandin E2, a major COX-2-derived product is a stimulator of angiogenesis and VEGF, thus inducing angiogenesis and promoting tumor progression (Fujita et al., 2002; van Moorselaar and Voest, 2002). Selective COX-2 inhibitors are potential therapeutic candidates to prevent PCa. Treatment with NS-938, a COX-2 inhibitor suppressed angiogenesis, induced apoptosis in tumor cells, decreased tumor MVD, inhibited tumor growth, and caused regression of existing tumors of PCa (Liu et al., 2000).

### Platelet-Derived Endothelial Cell Growth Factor

Platelet-derived endothelial cell growth factor (PDGF) belongs to a heparin-binding family of polypeptide growth factors A, B, C, and D. PDGF signals upon binding to class III receptor tyrosine kinases, PDGFRα, and PDGFRβ (Chen et al., 2013). PDGFRα exerts a pro-metastatic role by promoting PCa bone metastasis. Normal prostate tissue expresses low levels of PDGFα, while its expression is prominent in primary PCa and their skeletal metastases (Chott et al., 1999; Russell et al., 2009). PDGF natural ligands, in addition to multiple soluble factors contained within the human bone marrow, activates PDGFR-α which in turn activates the Akt pathway in metastatic prostate cells (Dolloff et al., 2007). The selective targeting of PDGFRα with monoclonal antibodies such as IMC-3G3 inhibits the growth of PCa cells at the skeletal level (Russell et al., 2009). Upon binding to their receptors (PDGFRα and PDGFRβ), PDGF ligands stimulate angiogenesis by upregulating VEGF-A production and regulating the perivascular cells proliferation (Laschke et al., 2006). The elevation in PDGFRβ activity is associated with the overexpression of VEGF-A and VEGFR-2 causes increased sprouting, pericyte coating, and vessel formation. Also, PDGF-B increases endothelial cell lineage commitment and restricted differentiation of hematopoietic precursors (Magnusson et al., 2007). VEGF-A upregulates the expression of endothelial PDGF-B, while FGF-2 enhances the expression of perivascular PDGFRβ. VEGF and FGF-2 together then stimulates perivascular cell recruitment and functional vasculature formation (Kano et al., 2005).

PDGF-B and PDGFRβ signaling mediates the recruiting and stabilizing of perivascular cells to form functional blood vessels (von Tell et al., 2006). Furthermore, PDGF-B regulates transcriptional activity of thrombomodulin in human vascular smooth muscle cells as it upregulates the transcription factor E26 transformation specific sequence-1 (Ets-1) in perivascular cells and endothelial cells (Lo et al., 2009). PCa cells, *via* PDGF production, induces the expression of PDGFR on tumor-associated endothelial cells and activates PDGFR by a paracrine action (Uehara et al., 2003). PDGFR expression is linked to tumor progression and bone metastasis. In the human multidrug-resistant PCa, the administration of paclitaxel and the tyrosine kinase inhibitor imatinib decreased the number of bone metastases, inhibited PDGFR phosphorylation in both endothelial cells and cancer cells, enhanced the apoptotic rate, decreased MVD, tumor size, and lymph node metastases (Kim et al., 2006).

### Other Angiogenesis Stimulating Factors

In PCa, intraductal grown tumor cells express angiopoietin-1 but not angiopoietin-2 while in the blood vessels close to the ducts, apical tumor cells express angiopoietin-1 and Tie-2. The tumor and intraglandular stromal cells in glandular PCa expresses both angiopoietin-1 and angiopoietin-2 (Wurmbach et al., 2000). The mural cell adhesion to endothelial cells is an essential step for the maturation of blood vessels. Angiopoietin-1 (Ang-1), a ligand for Tie2 receptor expressed on endothelial cells, aids in both mural endothelial cell adhesion which is needed for blood vessels to mature and in endothelial cell sprouting from preexisting vessels in the absence of mural cells (Satoh et al., 2008). In prostate tumors, Ang1 enhanced angiogenesis, tumor growth, and induced sprouting angiogenesis (Satoh et al., 2008). Besides, MVD, Angiopoietin-2 (Ang-2), and VEGF have been shown to contribute to the regulation of angiogenesis in different stages of PCa (Tesan et al., 2008).

Also, human prostate tissue expresses elevated levels of epidermal growth factor (EGF), transforming growth factor-α (TGF-α; Ching et al., 1993), and their corresponding EGFR that downregulates miR-1 and activates *TWIST1* causing an accelerated PCa bone metastasis (Chang et al., 2015). Furthermore, scatter factor/hepatocyte growth factor (Zhu and Humphrey, 2000), tissue factor (Abdulkadir et al., 2000), and *MUC1* (Papadopoulos et al., 2001) are other stimulators of angiogenesis expressed in PCa.

## Novel Molecular Pathways in PCa Microenvironment

In the era of precision and targeted therapy, it became crucial to develop a deep investigation and dissection of the several molecular pathways that are involved in PCa initiation, tumorigenesis, and metastasis (Daoud et al., 2016; Bahmad et al., 2020b; Cheaito et al., 2020).

Mahajan et al. revealed that ACK1/TNK2, a non-receptor tyrosine kinase, plays a key role in regulating the progression of CRPC. An activated ACK1 was present in the CD44+/PSA^−/lo^ cells. Furthermore, when PCa cells were treated with an ACK1 inhibitor such as (R)-9bMS; this has led to a diminished resistance to radiotherapy, an inability to establish spheres cultures and xenograft tumors in castrated mice from CD44+/PSA^−/lo^ cells. There was also significant apoptosis in prostate CSCs. All the aforementioned effects highly suggest that targeting ACK1 has an immense potential (Mahajan et al., 2018).

From another perspective, Aldahish et al. examined the calcitonin-calcitonin receptor (CT-CTR) pathway. When the CT-CTR pathway was induced in PC-3 and LNCaP cells, the adherence to collagen and the expression of CD133 and CD44 were significantly enhanced. The researchers also observed a potentiated tumorigenic and metastatic ability and an enhanced self-generation (Aldahish et al., 2019). One can conclude that the CT-CTR couple should be considered as a crucial target when it comes to developing therapies that hamper PCa development. Mitochondrial fission has also gained a considerable scientific attention when it comes to its role in regulating PCa. Civenni et al. (2019) demonstrated that genetically knocking down or pharmacologically inhibiting BRD4 blocked mitochondrial fission which depleted prostate CSCs and decreased their neoplastic potential.

Another notable molecular pathway that deserves further investigation is the estrogen alpha pathway (ERalpha). A recent study by Shen et al. (2019) demonstrated that CD49f+/ERalpha+ cells associated with basal stem-like and EMT characteristics have an enhanced metastatic potential. The study also revealed a potential downstream involvement of the NOTCH1 axis. Chen et al. (2020) recently demonstrated that the PCa microenvironment triggered the expression of Activin A *via* NF-kappaB stimulation. These high levels of Activin A also enhanced the development of an aggressive ALDH^hi^ phenotype through a Smad and ERK1/2 driven pathway. Finally, a very recent study by Hou et al. tried to investigate the regulation of prostate CSCs by thrombospondins 4 (THBS4). It was revealed that HBS4 silencing can hamper the development of PCa characteristics *via* blockade of the PI3K/Akt pathway (Hou et al., 2020).

A noteworthy subject that should be allocated a considerable research attention in the near future is the potential contribution of the stromal components in tumor neurosignaling. March et al. affirmed in their review that some neurotrophic growth factors and brain-derived neural progenitor cells has been previously shown to initiate tumor innervation. Not only would further investigations around this subject help in optimizing management and predicting prognosis, but they would also probably help in targeting the cancer-induced pain when bone metastasis occurs (March et al., 2020). Another subject that could be further dissected is the interaction of stromal components with neighboring muscles cells. In fact, one study revealed that the coculture of PCa cells with skeletal or smooth muscle cells boosts prostate CSC populations. This delicate interaction between muscle and cancer cells is accompanied by several processes: an increase in IL-4 and IL-13 secretion, an overexpression of annexin A5 and syncytin 1 and a noticeable fusion of cancer cells (Uygur et al., 2019).

In conclusion, several molecular pathways could be potentially targeted if prostate CSCs were chosen as a culprit. For example, with the promising results of the atypical adamantyl retinoid ST1926 in hampering PCSC growth, perhaps it would be interesting to further investigate the androgen independent molecular pathways that are involved in stemness and affected by retinoids (Bahmad et al., 2019).

## Laser-Capture Microdissection and Single-Cell RNA-Sequencing in PCa

The unique heterogeneity in the PCa microenvironment highlights a crucial need to employ technologies that can provide a subpopulation-specific analysis. In fact, such technologies would help researchers in thoroughly dissecting the intricate interactions between specific subpopulations and their surrounding microecology. Laser-capture microdissection (LCM) is a relatively novel technique where subpopulations of tissue cells can be extracted under direct microscopic visualization. This technique holds an immense potential given the multitude of downstream analyses that can follow (genotypic/transcriptomic profiling, signaling pathways discoveries, biomarker identification, and others; Espina et al., 2006). When it comes to PCa, LCM has been employed in a multitude of studies and several protocols have been established to generate downstream analyses (Lugli et al., 2015; Staunton et al., 2016). For instance, using LCM on N-cadherin positive patient-derived subpopulations of cells, Kolijn et al. (2018) demonstrated that during EMT, indoleamine 2,3-dioxygenase is overexpressed. Gregg et al. used LCM and microarray analysis to highlight the differential expression of several stromal and epithelial genes between healthy and neoplastic tissue. Interestingly, the study revealed that the expression of epithelial genes such as GATA2, FGFR-3, and WT1 was higher in PCa tissues than in healthy tissues. This was also the case with a multitude of stromal genes such as CXCL13, IGF-1, IGFBP3, CCL5, and FGF-2 (Gregg et al., 2010). Similarly, after coupling LCM with gene profiling studies, Tyekucheva et al. concluded that high-Gleason score tissues had a unique stromal gene signature that showcases immune-related pathways and bone remodeling. This stromal signature could also accurately predict metastatic potential (Tyekucheva et al., 2017).

Single-cell RNA-sequencing (scRNA-seq) would be another method that can potentially facilitate a precise subpopulation-specific analysis of the PCa microenvironment. In fact, many recent studies have supported the role of scRNA-seq in dissecting the heterogenous profile of the PCa microenvironment subpopulations. For example, Vickman et al. combined scRNA-seq of primary human prostate CAF with unsupervised clustering to produce subpopulation clusters based on the differential expression of genes. The study revealed that the CAF produced the CCL2 chemotactic chemokine which may be involved in the recruitment of immune cells to the tumor microenvironment and in tumor growth regulation (Vickman et al., 2020). Interestingly, Chen el al. used scRNA-seq to demonstrate that PCa can hijack the tumor microenvironment and modify T-cell transcriptomes. The uptake of tumor derived extracellular vesicles by T-cells leads to an increased expression of KLK3 and to a subsequent progression of micro-metastasis (Chen et al., 2021). All in all, it is possible to conclude that the development and the enhancement of novel technologies like LCM and scRNA-seq would greatly assist in deciphering the complex tumor microenvironment.

## Conclusion and Future Directions

A reactive tumor stroma has been shown to play a central role when it comes to tumor initiation and progression. In fact, it is known that prostate CSCs may be recruited to become carcinoma-associated fibroblasts or myofibroblasts. Multiple key regulators of this recruitment and transformation process have been identified. The delicate and complex interaction between the carcinoma itself and the stroma is meticulously controlled by the previously mentioned factors. One has to remember that the early birth of the reactive stroma, sometimes even during preneoplastic stages, and also failure of clinical trials on various inhibitors of angiogenesis and other processes pertaining to the prostate microenvironment in cancer, should prompt researchers to investigate more and look for clinically relevant potential targets before these stromal components exert its full-blown cancer-promoting effect (David and David, 2012). More importantly, circulating plasma/urine biomarkers are of utmost importance and might indeed aid in the diagnosis, treatment selection, and response assessment of PCa in the near future. Additionally, one must acknowledge that many lessons could be learned from the drawbacks of previous anti-angiogenic drug trials and, therefore, the scientific efforts should be directed to paths that avoid previously committed mistakes. Additionally, the unmet need of fully exploiting the potential of biomarkers other than PSA should be addressed since over-diagnosing and over-treating patients are two crucial problems. For example, the prostate health index (PHI) and the prostate cancer antigen 3 (PCA3) are two more specific FDA-approved biomarkers. Although PHI is significantly cheaper, the non-invasive 4Kscore blood test also showed a potential to forecast clinically significant PCa and, therefore, this would ultimately result in a decrease in unneeded biopsies. Other urine liquid biopsy tests such as Exosome Dx and Select MdX showed a notable potential too, but further studies are needed before establishing a full clinical integration. One could additionally conclude that more head-to-head trials are needed to reach a complete acceptance of a certain marker (Becerra et al., 2020). Collectively, remodeling the neoplastic environment constitutes a crucial step that propels tumor invasiveness. This process is heavily dependent on the penetration of the extracellular matrix, restructuring of fibrillar components, and the carcinoma-stroma interaction.

## Author Contributions

HB: investigation, methodology, writing – original draft preparation, writing – reviewing and editing, visualization, and validation. MJ, JA, and MM: investigation, methodology, writing – original draft preparation, writing – reviewing and editing, and validation. TS and DM: investigation, writing – reviewing and editing, visualization, and validation. MA-S: investigation, project administration, supervision, writing – reviewing and editing, visualization, validation, and funding acquisition. WA-K: conceptualization, project administration, supervision, writing – reviewing and editing, validation, visualization, and funding acquisition. All authors contributed to the article and approved the submitted version.

### Conflict of Interest

The authors declare that the research was conducted in the absence of any commercial or financial relationships that could be construed as a potential conflict of interest.

## Figures and Tables

**Table:** 

Term	Definitions
PCa	Prostate cancer
ECM	Extracellular matrix
MMP	Matrix metalloproteinases
MT1	Membrane type 1-matrix
BTF3	Basic transcription factor 3
CSC	Cancer stem cell
rBC2LCN	Recombinant N-terminal domain of BC2L-C lectin
Skp2	S-Phase Kinase Associated Protein 2
BMI1	B lymphoma Mo-MLV insertion region 1 homolog
Sox2	SRY-box 2
ALDH1	Aldehyde dehydrogenase isoform 1
MUC1	Mucin 1
FGF	Fibroblast growth factor
CRPC	Castration resistant prostate cancer
TNF	Tumor necrosis factor
NF-κB	Nuclear factor-*κ*B
IL	Interleukin
VEGF	Vascular endothelial growth factor
miR	Micro-RNA
Wnt	Wingless homologs
LncRNA	Long non-coding RNA
PTX3	Pentraxin 3
CUL4B	Cullin 4B
HIF-1	Hypoxia-inducible factor 1
TGF-β	Transforming growth factor beta
CTGF	Connective tissue growth factor
COX-2	Cyclooxygenase-2
PDGF	Platelet-derived endothelial cell growth factor
Ang-1	Angiopoietin-1
EGF	Epidermal growth factor
TWIST1	Twist Family BHLH Transcription Factor 1
TGF-α	Transforming growth factor-*α*
THBS4	Thrombospondins 4
